# Flexible Pressure Sensor Based on PVDF Nanocomposites Containing Reduced Graphene Oxide-Titania Hybrid Nanolayers

**DOI:** 10.3390/polym9020033

**Published:** 2017-01-26

**Authors:** Aisha Al-Saygh, Deepalekshmi Ponnamma, Mariam AlAli AlMaadeed, Poornima Vijayan P, Alamgir Karim, Mohammad K. Hassan

**Affiliations:** 1 Materials Science & Technology Program (MATS), College of Arts & Sciences, Qatar University, Doha 2713, Qatar; 200655132@student.qu.edu.qa; 2 Center for Advanced Materials, Qatar University, Doha 2713, Qatar; deepalekshmi@qu.edu.qa (D.P.); poornimavijayan@qu.edu.qa (P.V.P.); mohamed.hassan@qu.edu.qa (M.K.H.); 3 College of Polymer Science and Polymer Engineering, Akron Functional Materials Center (AFMC), The University of Akron, Akron, OH 44325, USA; alamgir@uakron.edu

**Keywords:** hybrid additives, nanocomposite, dielectric, relative resistance, synergy

## Abstract

A novel flexible nanocomposite pressure sensor with a tensile strength of about 47 MPa is fabricated in this work. Nanolayers of titanium dioxide (titania nanolayers, TNL) synthesized by hydrothermal method are used to reinforce the polyvinylidene fluoride (PVDF) by simple solution mixing. A hybrid composite is prepared by incorporating the TNL (2.5 wt %) with reduced graphene oxide (rGO) (2.5 wt %) synthesized by improved graphene oxide synthesis to form a PVDF/rGO-TNL composite. A comparison between PVDF, PVDF/rGO (5 wt %), PVDF/TNL (5 wt %) and PVDF/rGO-TNL (total additives 5 wt %) samples are analyzed for their sensing, thermal and dielectric characteristics. The new shape of additives (with sharp morphology), good interaction and well distributed hybrid additives in the matrix increased the sensitivity by 333.46% at 5 kPa, 200.7% at 10.7 kPa and 246.7% at 17.6 kPa compared to the individual PVDF composite of TNL, confirming its possible application in fabricating low cost and light weight pressure sensing devices and electronic devices with reduced quantity of metal oxides. Increase in the β crystallinity percentage and removal of α phase for PVDF was detected for the hybrid composite and linked to the improvement in the mechanical properties. Tensile strength for the hybrid composite (46.91 MPa) was 115% higher than that of the neat polymer matrix. Improvement in the wettability and less roughness in the hybrid composites were observed, which can prevent fouling, a major disadvantage in many sensor applications.

## 1. Introduction

Sensor technology is one of the widely used technologies for applications in the industry and medicine. It can be used to measure pressure, temperature, quality, and amount of energy, and to monitor health. High performance electrochemical devices such as sensors require good semiconductor properties, lightweight nature and enhanced energy storing capabilities. Various types of sensors have been fabricated from polymer matrices such as pressure [1], thermal/infrared [2], vapor [3], humidity [3], gas [4,5], electrical [4] and temperature/thermal sensor [5]. The most important parameters in sensor technology are response time and sensitivity [6].

Conducting Polymer nanocomposites (CPC) are used for sensor applications mainly as signal transducers. CPCs are specifically attractive for building sensor devices, as they possess unique physical properties, high surface area, and small dimensions. A piezoelectric material such as polyvinylidene fluoride (PVDF) is used for developing sensors as it can transfer the mechanical loads into electrical signals. PVDF has a crystalline phase [7] that can be effectively utilized for pressure sensing applications. The presence of β phase (crystalline region) in this polymer is responsible for its piezoelectric response [8]. Furthermore, PVDF is a pyroelectric material [9] and is non-toxic, flexible, and lightweight [10].

Pi et al. [11] synthesized a flexible piezoelectric nanogenerator thin film of (polyvinylidene fluoride-*co*-trifluoroethylene) prepared by spin coating. The flexible film can convert the mechanical force to electrical energy. The electrical output was obtained by applying a mechanical load and it was demonstrated to fabricate a high performance generator. The fabricated nanogenerator exhibited a short circuit current of 58 nA and open circuit voltage up to 7 V with the current density of 0.56 μA/cm^2^. A novel PVDF/perfluorooctyl triethoxysilane (PFOES)-rGO piezoelectric film was also prepared by solution mixing to be used as a modifying agent [12]. Compared to neat PVDF, the piezoelectric and dielectric constant of the PVDF/PFOES-rGO composite were improved by 80.9% and 379%, respectively [12]. In addition, the PVDF nanocomposites are highly demanded due to their low cost and lightweight applications [13,14]. A high sensitivity tracile sensor based on piezoelectric PVDF polymer was prepared by Spanu et al. [13], which showed the ability to detect pressure as low as 300 Pa with good reliability.

The addition of nanofillers is common to increase the β phase in PVDF composites, further leading to the increase in piezoelectric property. Carbon based nanomaterials gained much interest due to their compatibility, high surface area, prominent mechanical properties and electron transport [14]. Graphene oxide (GO) and graphene have the potential to modify polymer matrix and be used as a sensor [14,15]. GO specifically has excellent mechanical properties, high surface area, and has oxygen-containing functional groups such as carboxyl, carbonyl, epoxide and hydroxyl groups in its structure. GO can be incorporated with polymer matrices to improve the physical properties and mechanical strength [16]. It was found that the addition of 0.1 wt % of GO to PVDF matrix increases the β phase content [17]. In another study, multiwalled carbon nanotube (MWCNT) was added to PVDF, where an increase in β phase content was achieved with improved piezoelectric property [18]. This was because of the enhanced polymer-filler interfacial interactions. Recent reports show the applicability of a variety of metal oxides as reliable sensing materials at room temperature with high sensitivity and faster response [19,20,21]. One interesting example is the titanium dioxide (TiO_2_) which emerged as an attractive metal oxide additive for electronic devices fabrication because of its low cost, abundance, environmentally benign, and structural stability characteristics [22].

In this work, we report a new novel flexible PVDF hybrid pressure sensor with good mechanical properties. The material is prepared by the addition of a hybrid combination of rGO and metal oxide to the PVDF. Titania nanolayers (TNL) were synthesized and used in this work based on nanostructured TiO_2_. The high aspect ratio and transparency in addition to the improved photocatalytic performance were already established for the TNL [23,24]. Since the two-dimensional sheets of both TNL and rGO can create a tortuous path, the filler network formation within PVDF, to ensure conductivity, will be comparatively easy. The good dispersion of TNL and rGO enhanced the mechanical strength and dielectric properties in addition to enhancing the electrical sensitivity. To the best of our knowledge, this is the first report that shows the fabrication of a low cost composite by making use of these hybrid additives. We adopted the solution casting method in preparation of the nanocomposites which is simple and low cost when compared to the other composite fabrication techniques like melt mixing or in situ polymerization [25,26].

## 2. Experimental Details

### 2.1. Materials

PVDF with molecular weight (*M*_W_) 180,000 was purchased from Sigma Aldrich (St. Louis, MO, USA). The chemicals used for the preparation of GO and TNL, H_2_SO_4_, H_3_PO_4_, graphite, KMnO_4_, H_2_O_2_, NaOH and HCl were also obtained from Sigma Aldrich. The solvents, acetone, DMF, ethanol and ether were purchased from BDH chemicals (Atlanta, GA, USA). The TiO_2_ (anatase) nanopowder with a particle size of 15 nm was supplied by Nanostructured & Amorphous Materials, Inc. (Houston, TX, USA). All chemicals were used without any further purification.

### 2.2. Methods of Preparation

#### 2.2.1. Preparation of Graphene Oxide

**Figure 1:**
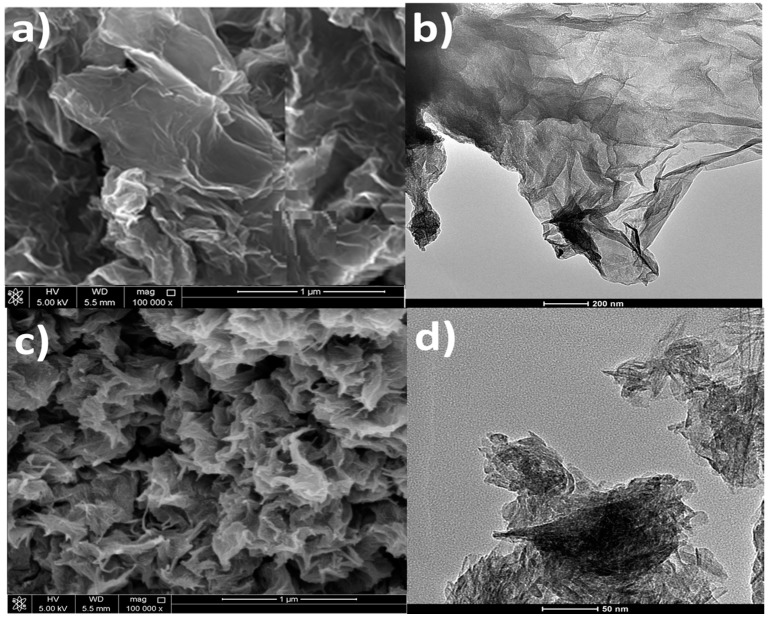
Morphology of reduced graphene oxide (rGO) (**a**,**b**) and titania nanolayers (TNL) (**c**,**d**). Pictures on left are scanning electron microscopy (SEM) while those on right are transmission electron microscopy (TEM).

The improved graphene oxide synthesis was applied to synthesize GO from the graphite precursor [27]. In this process, a mixture of concentrated H_2_SO_4_ and H_3_PO_4_ in the ratio of 9:1 was added to a graphite (3.0 g, 1 wt equiv)/KMnO_4_ (18.0 g, 6 wt equiv) mixture. The whole mixture was then heated to 50 °C with stirring for 12 h. Cooling was then arranged by adding ice (400 mL) with 30% H_2_O_2_ (3 mL) to neutralize the excess KMnO_4_. A metallic US standard testing sieve was then used to filter out the unreacted large graphite plates. The filtrate was centrifuged and the remaining solid material was washed in succession with water, 30% HCl, ethanol and ether and then dried. The prepared GO was thermally treated in oven at 50 °C for 5 h for reduction purpose. The dried rGO powder was analyzed for its sheet like morphology as shown in the scanning electron microscopy (SEM) and transmission electron microscopy (TEM) images in Figure 1a,b, respectively. The thickness of rGO was around 1.5 nm.

#### 2.2.2. Preparation of Titania Nanolayers

TNL was synthesized from TiO_2_ precursor by hydrothermal method [28]. In this technique, 1.2 g of TiO_2_ nanopowder was treated with 20 mL of 10 N NaOH solution in a Teflon beaker and stirred vigorously for 15 min. The mixture was then transferred into a Teflon lined autoclave and heated in a preheated oven at 130 °C for 10 h. The obtained precipitate was washed with deionized water. The washed precipitate was dipped in 0.1 M HCl solution for 30 min and washed again with HCl solution and distilled water until the solution became neutral (pH = 7). Finally, the synthesized powder was dried in the oven and morphology was checked. The sheet like structure obtained for this nanomaterial is shown by the SEM and TEM images in Figure 1c,d, respectively. The size of TNL obtained was around 2 nm.

#### 2.2.3. Preparation of PVDF Nanocomposites

**Figure 2:**
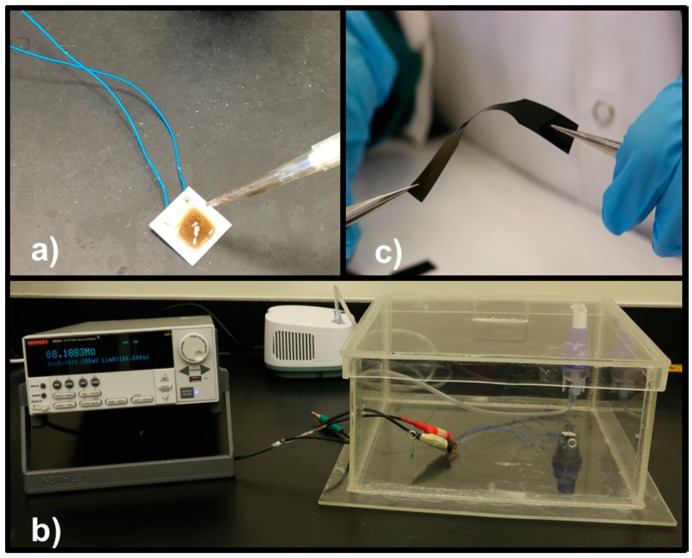
Samples coating on the interdigitated electrode (**a**); and the setup for the pressure sensing experiment under controlled conditions (**b**). A picture showing the flexibility of the polyvinylidene fluoride/reduced graphene oxide-titania nanolayers (PVDF/rGO-TNL) composite sample (**c**).

rGO and TNL fillers in specific percentages (Table 1) were dissolved in the DMF/acetone mixture separately by bath sonication for 2 h followed by magnetic stirring for 12 h at 500 rpm. This ensured maximum level of filler dispersion in the solvent mixture. In the next step, the filler dispersions in 2.5 and 5 wt % were mixed with the PVDF solution and the whole mixture was magnetically stirred overnight at 500 rpm. After mixing, the solutions were casted on petri dishes and kept in the oven for 10 h at 70 °C to have nanocomposite films. Later, the films were made homogeneous by hot pressing at 170 °C for 2 min (Figure 2b). The prepared PVDF composites are shown in Table 1.

### 2.3. Characterization Techniques

The sample morphology was analyzed using scanning electron microscope (SEM) (JEOL JCM-6000Plus Versatile Benchtop, Akishima, Tokyo, Japan), (FEI Nova NanoSEM 450, Hillsboro, OR, USA); TEM (FEI Tecnai™ transmission electron microscope, Hillsboro, OR, USA) and atomic force microscope (AFM) (MFP-3D AFM, Asylum Research, Santa Barbara, CA, USA). PerkinElmer Spectrum 400 spectrophotometer (Waltham, MA, USA) was used to record the Fourier transformation infrared spectra (FTIR) of the samples in the range 400–4000 cm^−1^ with a resolution of 2 cm^−1^. X-ray diffraction test was performed using X-ray Diffractometer (Empyrean, Panalytical, Nottingham, UK) within the 2θ range 5° to 30°. The contact angle measurements were done using optical contact angle (OCA 35, data physics, Filderstadt, Germany) following the sessile drop principle. The instrument was connected with automatic image acquisition and computation software to calculate the corresponding contact angles. For each sample, the measurements were done three times and an average was taken.

The mechanical test was carried out using universal testing machine (Lloyd 1KN LF Plus, AMETEK, Inc., Bognor Regis, UK) at a speed rate of 5 mm/min. The dimensions of specimen were prepared according to ASTM D882-10 [29]. The dynamic mechanical behavior of the PVDF nanocomposites was done using dynamic mechanical analyzer (RSA G2, TA Instruments, New Castle, DE, USA). For the differential scanning calorimetric experiments, Differential scanning calorimeter (DSC 8500, PerkinElmer, Waltham, MA, USA) was used. Dielectric properties were recorded by means of Novocontrol GmbH Concept 40 Broadband Dielectric Spectrometer (Novocontrol Technologies GmbH, Montabaur, Germany) at room temperature and over a broad range of frequency from 10^−1^ to 10^6^ Hz. Sample discs of 2 cm diameter were sandwiched between two gold coated copper electrodes of same diameter. The discs were transferred to the instrument for data collection.

**Figure 3:**
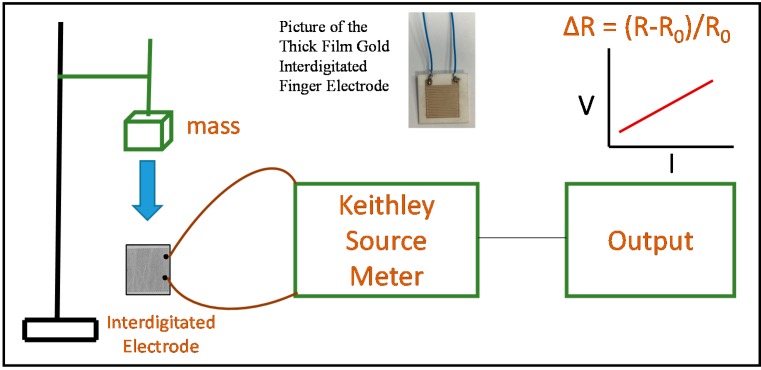
Schematic representation of the experimental setup for the pressure sensing experiment. A picture of the interdigitated electrode used in the measurements is also shown.

The sensing experiments were done by measuring the electrical resistance variation of the composites using a Keithley 2400 Series Source Meter (Tektronix, Beaverton, OR, USA). The measurements were performed at room temperature and 40%–60% relative humidity. Samples solution were coated on the electrode active area (1 cm × 1 cm) and dried to form thin films of ≅40 μm thickness, as illustrated in Figure 2a. The set up for the whole sensing experiment is shown in Figure 2c and is schematically presented in Figure 3. The sensors were excellent in its performance while detecting high pressures of the order of 10^3^ Pa. Due to this reason, the responses at three particular pressures, 5, 10.7 and 17.6 kPa are demonstrated here with applied pressures at every 10 s under ambient conditions. The electrical resistance upon introducing the stimulus can be varied quantitatively according to Equation (1), where (*A*_R_) is the relative resistance, (*R*) the final resistance and (*R*_0_) the initial resistance.

(1) $$A_{R\;}=\;\frac{R\;-\;R_{0}}{R_{0}}\times \;100$$

## 3. Results and Discussion

### 3.1. Morphology and Structure

**Figure 4:**
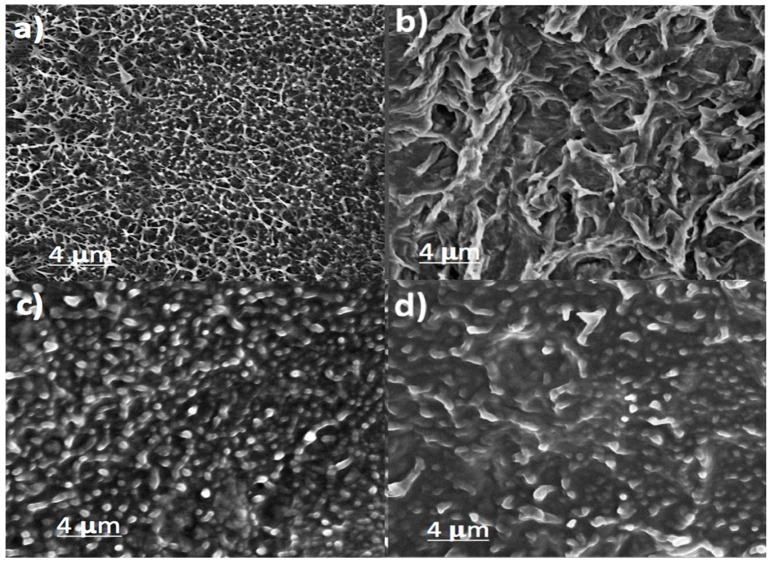
SEM images of cross-sections of samples: (**a**) PVDF; (**b**) PVDF/rGO; (**c**) PVDF/TNL; and (**d**) PVDF/rGO-TNL.

Figure 4 shows the morphology of the PVDF and its nanocomposites as observed by the SEM. The cryo-cut samples in liquid nitrogen were used for this analysis and the cross-sectional area is observed through the microscope. Neat PVDF shows a plain type morphology (absence of fillers) as shown in Figure 4a. The rGO and TNL are visible as nanolayers in the cross sectional area of the samples shown in Figure 4b,c, respectively. For the hybrid PVDF/rGO-TNL, the nanolayers are homogeneously distributed due to the functional group interaction between both nanomaterials (Figure 4d). The oxygen content in the functional groups that present on the surface of rGO can interact with the titania nanolayers by hydrogen bonding as well as Van der Waals interactions and this result in good dispersion of the fillers inside the PVDF medium. Similar interaction was reported by Xu et al. [30] for GO nanosheets with starch granules. In their work, starch surfaces were covered by the large surface of the GO nanosheets, which have high surface energy. The additives in this case with enhanced interfacial interactions had uniform dispersion in the polymer matrix.

**Figure 5:**
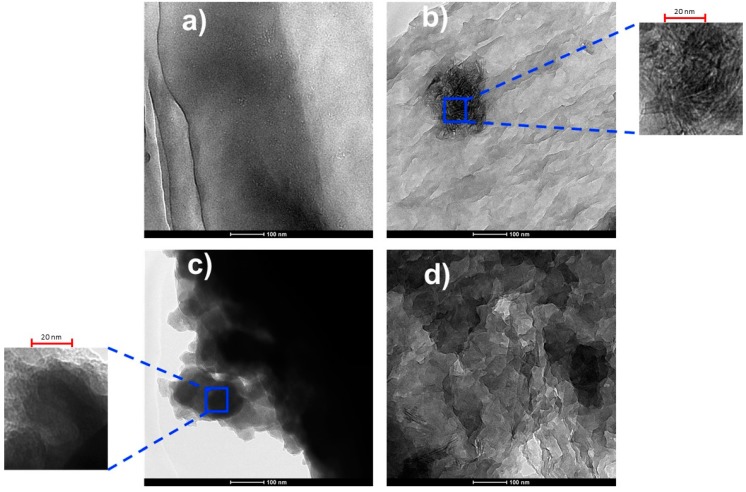
TEM images of surface of: (**a**) PVDF; (**b**) polyvinylidene fluoride/titania nanolayers (PVDF/TNL); (**c**) PVDF/rGO; and (**d**) PVDF/rGO-TNL.

Figure 5 shows the TEM images of the surface of PVDF, PVDF/TNL, PVDF/rGO and PVDF/rGO-TNL. These images display a homogenous surface of hybrid composite (Figure 5d), compared with PVDF/TNL (Figure 5b) and PVDF/rGO (Figure 5c) that shows some agglomeration. Agglomeration occurring in PVDF/rGO and PVDF/TNL are represented as focused high-resolution images as well. Such homogenous image indicates that rGO sheets and TNL were fully dispersed in PVDF matrix. The good dispersion is due to the Van der Waals interaction between rGO and TNL, higher than those between individual rGO sheets [30]. The TEM results thus further substantiate the observation from SEM studies.

**Figure 6:**
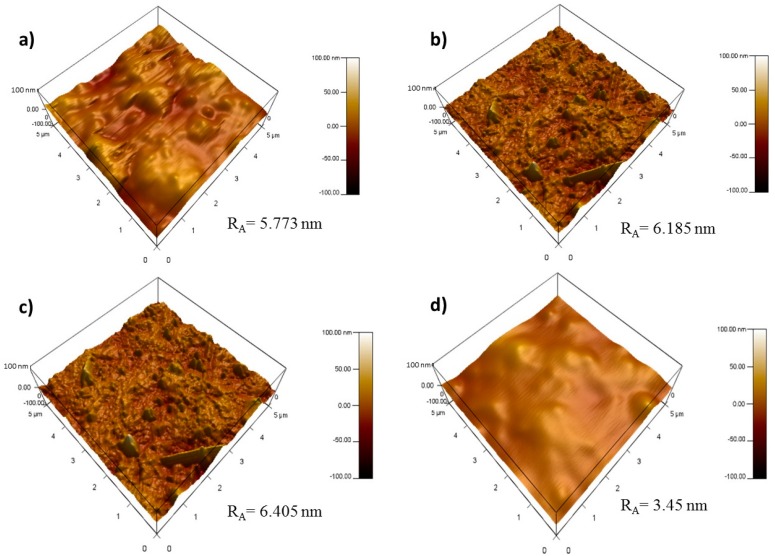
AFM images of surface of: (**a**) PVDF; (**b**) PVDF/rGO; (**c**) PVDF/TNL; and (**d**) PVDF/rGO-TNL.

Figure 6 illustrates the AFM images of PVDF, PVDF/TNL, PVDF/rGO and PVDF/rGO-TNL. The average roughness of PVDF is 5.773 nm. With the addition of rGO and TNL, the average roughness increased to 6.185 nm (Figure 6b) and 6.405 nm (Figure 6c), respectively. The average roughness decreased to 3.45 nm in the hybrid composite, which indicates smoother surface as seen in Figure 6d. This smooth surface reflects the homogeneous distribution of the fillers. This could be also due to the fuse of TNL on rGO, similar improvement of the smoothness was reported by Dai et al. [31] for the Al_2_O_3_ with polyurethane and PVDF. One important advantage of such reduction in roughness is its critical applicability in improving the anti-fouling properties of this composite.

**Figure 7:**
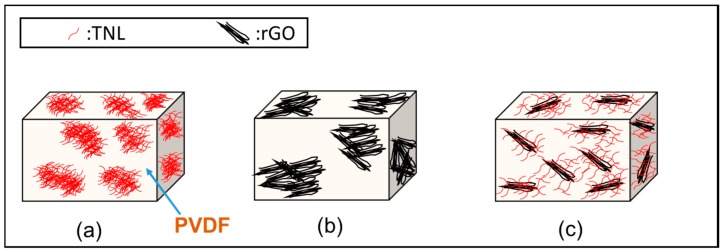
Schematic representation of the dispersion of nanoparticles in: (**a**) PVDF/TNL; (**b**) PVDF/rGO; and (**c**) PVDF/rGO-TNL.

Based on different microscopic investigation, it is clear that the nanolayers of rGO and TNL create a well-distributed path within the PVDF in the hybrid composite PVDF/rGO-TNL. Moreover, the individual nanomaterials at 5 wt % cause agglomeration in the PVDF/rGO and PVDF/TNL composites. This mode of dispersion is represented in Figure 7.

**Figure 8:**
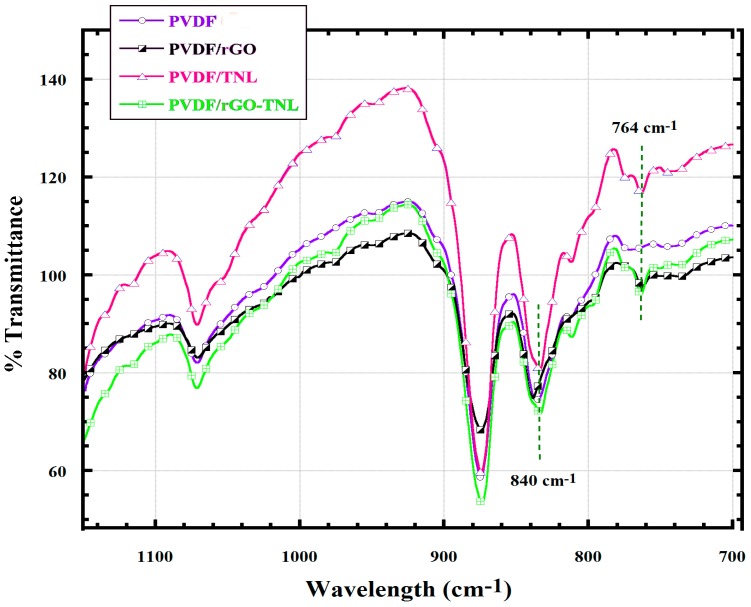
FTIR spectra of neat PVDF and its PVDF/rGO, PVDF/TNL, and PVDF/rGO-TNL composite samples.

In order to check the structural properties, especially the functional groups presented in the material and the chemical modifications, FTIR spectroscopy of samples were taken. Figure 8 shows the FTIR spectra for the neat PVDF and PVDF composites.

All the peaks observed in the graph come from the C=C stretching, bending and C–H bending vibrations. The dipole–dipole interaction in PVDF/rGO-TNL is also possible according to the FTIR spectrum. This indicates that there is no chemical bond formation between the filler and polymer, but just physical interaction exists. An interesting factor in the FTIR spectrum is the absence of the characteristic peaks for the C=O, –OH, –COOH etc. functional groups usually associated with the rGO. Following solution mixing, the samples were made homogeneous by hot pressing at 170 °C and this might have again further reduced the functional groups present in rGO. All samples show peaks related to PVDF at around 1000 cm^−1^ representing –CH_2_ rocking and at 800 cm^−1^ representing –CF_2_ asymmetric stretching [32]. The FTIR data indicated peak for β phase at 840 cm^−1^ and alpha phase at 764 cm^−1^ [33]. FTIR is commonly used to identify the β crystalline phases in a polymer by the following equation [7]:

(2) F(β)=ΧβΧα+Χβ= Aβ(KβKα)Aα+Aβ

where *K*_α_ and *K*_β_ are the absorption coefficients at the particular wavenumber. *K*_α_ is 6.1 × 10^4^ cm^2^/mol and *K*_β_ is 7.7 × 10^4^ cm^2^/mol. *X*_α_ and *X*_β_ are mass fraction of α and β crystalline phases. *A*_α_ and *A*_β_ are the area of absorption bands at 764 and 840 cm^−1^. The relative fraction for β phase using the Equation (2) are 70.37%, 72.73%, 73.5% and 75.68% for PVDF, PVDF/rGO, PVDF/TNL, and PVDF/rGO-TNL, respectively. The results indicate that β phase increased with hybrid PVDF/rGO-TNL additives compared to neat PVDF. The improvement in the β phase (more chains are rearranged at the same side of the fluorine atoms) is due to the good interaction of the hybrid additives with the fluorine in the polymer matrix. Similar improvement was reported for rod like cellulose in a PVDF matrix due to the presence of OH groups [34].

**Figure 9:**
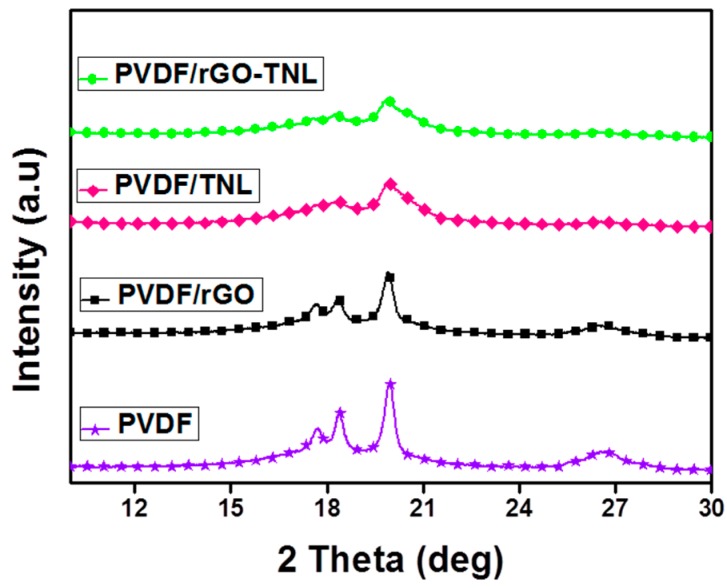
X-ray diffraction pattern of PVDF, PVDF/rGO, PVDF/TNL, and PVDF/rGO-TNL.

X-ray diffractograms for all the composites-PVDF, PVDF/rGO, PVDF/TNL and PVDF/rGO-TNL are shown in Figure 9. When an X-ray is allowed to fall on a crystalline material, the incident beam interacts with the aligned atoms and thus diffraction occurs. Intensity of diffracted radiations change due to the coherent interference of the individual atoms. The diffraction data in terms of peak position and intensity yield information on the overall chemistry of the samples. Here, all samples show similar peak appearances due to the presence of semi crystalline PVDF in all of them. Eggedi et al. reported that the peaks associated with α phase of PVDF at 2θ of 18.6°, 20.3° and 27°, whereas the β phase is found as sharp peak at 20.6° [35]. In our results it can be observed that α peaks are at 18.4°, 19.9° and 26.6° and the β phase being hidden inside the α peak at 19.9°. It has been observed that the areas of all α peaks were reduced or vanished in the hybrid composite. The influence of nanoparticles on the XRD patterns depends on the nature of the particles [36], the hybrid additives approximately erased the α peaks, and caused the β peak to be broadened. This indicates that the nanoadditives may interrupt the packing, which will also be seen in DSC results.

### 3.2. Surface Properties

The dependence of the water contact angle on the surface of the nanocomposite is calculated and tabulated in Table 1. For the neat PVDF, the contact angle is 99.7°, which also reported elsewhere [37]. The water contact angle decreased for PVDF/rGO to 95.5° due to the partial hydrophilicity of the rGO platelets because of the presence of OH group on its surface. For the PVDF/TNL, the contact angle has the approximate same value of the neat PVDF. For the PVDF/rGO-TNL an increase in the contact angle to 114.5° is observed. This can be attributed to the synergistic effect of the fillers producing a network like structure within the composite and the surface as confirmed by TEM and AFM results, such concentrated filler networks can make the surface more hydrophobic. The contact angle is also related to the interaction parameter; the higher the interaction occurring within the system, the more resistant it will be towards the liquid contacting the surface [38].

### 3.3. Mechanical and Dynamic Mechanical Properties

The tensile test was performed to examine the mechanical properties of the samples. The obtained values for tensile strength, Young’s modulus and elongation at break are shown in Table 2.

**Figure 10:**
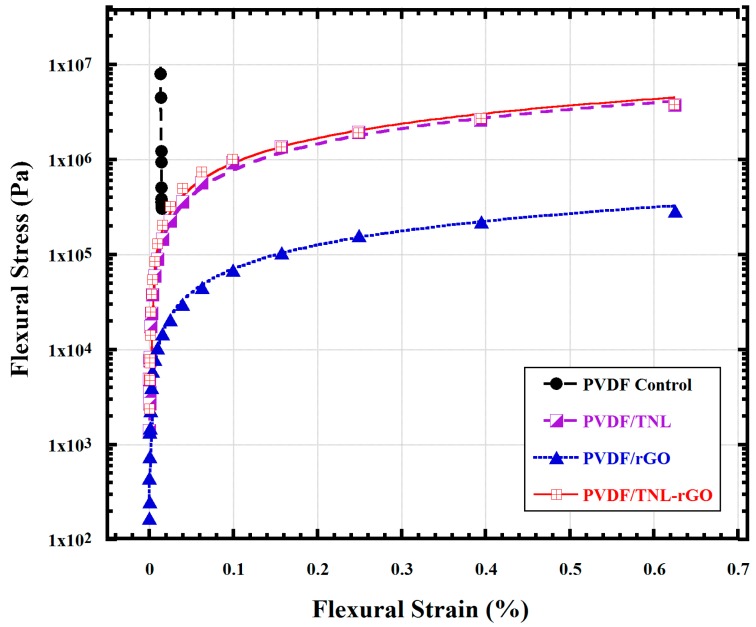
Three-point bending test for neat PVDF and its nanocomposites.

Nano additives increased the tensile strength and Young’s modulus. Among the composites, tensile strength and Young’s modulus for the hybrid composite PVDF/rGO-TNL was 2 and 3.6 times, respectively, higher than that of the neat PVDF. The elongation at break was reduced with the addition of the filler and reached its minimum value of 4% for the hybrid composite, compared to 16% for the neat PVDF sample. This indicates the brittle behavior of the hybrid composite. The improvement in the mechanical properties can be attributed to the increase in the β crystallites in the matrix (confirmed by FTIR measurements). Al-Maadeed et al. [39] reported the effect of increase in crystallinity on modification of the tensile strength in a polymer. In addition, Issa et al. [7] mentioned that increase in crystallinity and crystal orientation increase the tensile strength of the PVDF. Mechanical integrity enhancement of the composites was further supported by the three-point bending test, which was conducted according to the ASTM standard D790, Figure 10. The control PVDF breaks before yielding. While, the composite samples neither yields nor breaks as the strain increases. The results reflect the *flexibility* of the composite samples, as they yield not breaking as the flexural strain increases.

Enhanced mechanical properties of our hybrid nanocomposite can be correlated with two factors: (1) the interfacial interactions existing between the rGO and TNL as well as the fillers and the PVDF; and (2) the synergistic effect of the fillers. It is well established that the synergistic effect can enhance the mechanical properties of the sample [40]. The filler content in the PVDF/TNL and PVDF/rGO (5 wt %) is considered high and possibility of nanofiller agglomeration in the matrix could cause the reduction of the elongation at break.

**Figure 11:**
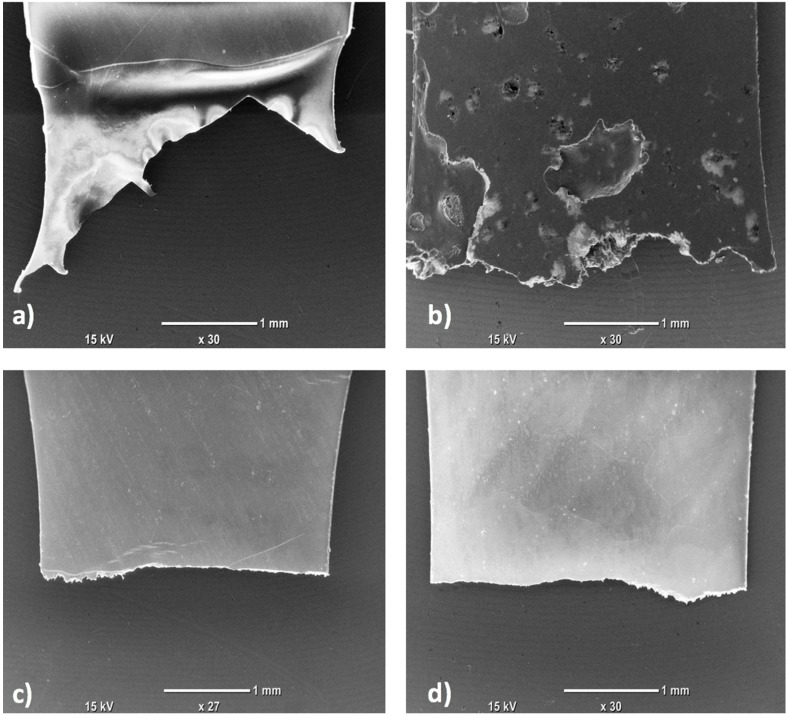
SEM images of surface of tensile test specimen cut: (**a**) PVDF; (**b**) PVDF/rGO; (**c**) PVDF/TNL; and (**d**) PVDF/rGO-TNL.

The images of the fractured surface after mechanical deformation by tensile test are shown in Figure 11. It clearly shows a ductile fracture for PVDF as the fracture surfaces of the tensile test specimens cut showed deep cavities (Figure 11a). The fracture surface of PVDF/rGO shows a micro crack as indicated in Figure 11b. Figure 11c shows fewer cavities in the fracture surface. The ductility reduction in Figure 11d could be caused by reduction in ductile fracture. These results are also confirmed by the tensile test values from Table 2.

**Figure 12:**
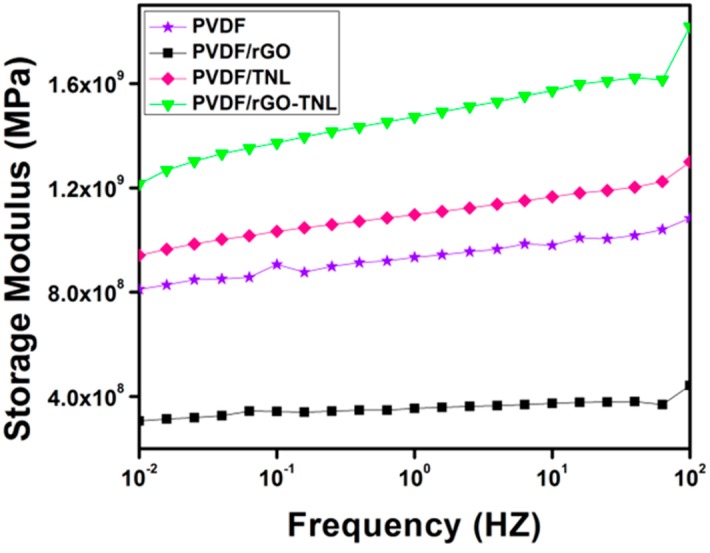
Variation in storage modulus with frequency for PVDF and its nanocomposites.

Figure 12 shows the variation in storage modulus of the samples as functions of frequency. The hybrid composite PVDF/rGO-TNL has higher storage modulus compared to other samples. This indicates that this composite has good property in storing energy for piezoelectric applications [40]. The improvement in the storage modulus can be attributed to the improvement in the β crystal formation [41], or the better interaction between the matrix and the filler. It is also important to note that the high rigidity of the additives contributes to this improvement, which was also reported elsewhere [7].

### 3.4. Thermal Properties

DSC was performed to study the changes in melting enthalpy (Δ*H*_m_), melting temperature (*T*_m_), and degree of crystallinity (*X*_c_) as shown in Table 3. Values of *X*_c_ were calculated using the following Equation (3).

**Figure 13:**
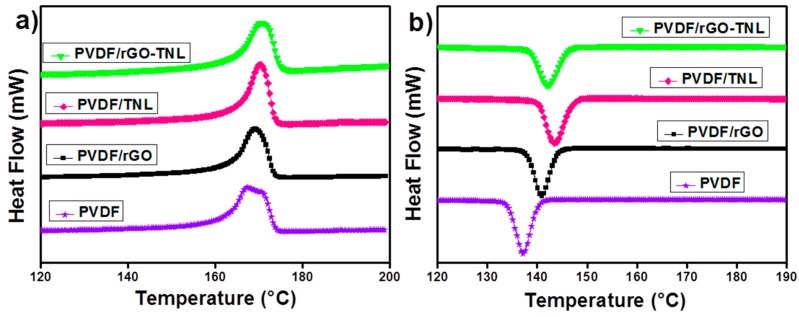
DSC analysis for PVDF and its nanocomposites: (**a**) Melting curve; and (**b**) Crystallization curves.

(3) Xc=∆Hm∆Hm0

where $∆H_{m}$ and $∆H_{m}^{0}$ are the melting enthalpies of the composite and neat PVDF, respectively. The melting enthalpy for pure PVDF is 105 J·g^−1^ [42]. Figure 13a shows the melting peak of PVDF has two shoulders, indicating two types of crystallites [43] with two different lamellae thicknesses [44]. This peak shifts to higher temperatures with the addition of the fillers.

The cooling peaks in Figure 13b are also affected by the additives. The crystallization temperature increased by 5 °C for rGO addition and around 7 °C for TNL addition. The hybrid additives increased the crystallization temperature by 6 °C. This increase in temperature indicates the easiness of the crystallization with the addition of these additives. These additives act as nucleating agents that encourage the formation and growth of the crystallites in the polymer [45]. Table 3 shows the decrease in crystallinity for PVDF/rGO-TNL than PVDF. The increase in the melting points, which indicates increase in lamellae, does not guarantee the increase in the crystallinity percentage in the polymer. The crystallinity percentage depends on other factors such as length, distribution and curvature [46]. According to XRD results, there is a reduction (erase) in the α phase and change in the β phase peak. Dai et al. [31] reported similar study that additives that were distributed uniformly in the matrix interrupted the packing of chains and decreased the crystallinity.

### 3.5. Dielectric Properties

Dielectric spectroscopy is one of the methods that addresses electrical polarizability and molecular dynamics in polymers and nanomaterials [47]. Under applied electric field, the polarizable elements or material dipoles interact and oscillates at different frequencies (ω) and certain temperatures. The response of the material to the applied electric field is usually conveyed in terms of the complex dielectric permittivity [48].

(4) $$\varepsilon^{*}\left(\omega \right)=\;\varepsilon \prime \left(\omega \right)-i\;\varepsilon \prime \prime \left(\omega \right)$$

where ε′ is the real permittivity which reflects material polarizability due to distortion of delocalized electron distributions, interfacial polarization (internal or sample/electrode) or dipole reorientation. ε″ is loss permittivity and is related to the energy dissipated per cycle during any of these processes. ω is the angular frequency (2π*f*). Broadband dielectric spectroscopy is considered a powerful tool for probing the molecular dynamics of polymer chains as important information regarding these processes can be deduced over wide frequency (milli to mega) hertz and temperature regions [47].

**Figure 14:**
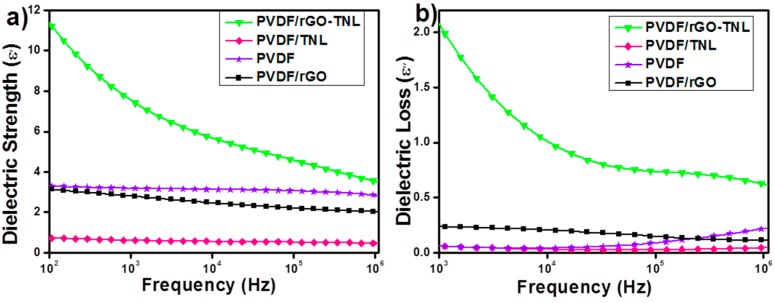
Dielectric permittivity storage (**a**); and loss (**b**) of PVDF control and its composite samples with variable nanoparticle fillers.

Figure 14 shows the frequency (*f*) dependence of the dielectric permittivity storage (ε′) and loss (ε*ʺ*). The Dielectric permittivity storage (ε′) is constant for the PVDF and one type of additive but it decreases as *f* increases for the PVDF/rGO-TNL sample. Decrease in ε′ with increasing *f* is due to an increasing inability of the time scale of the dynamics of polarization to be apprehended within increasingly shorter signal time scales, that is, decreasing 1/2*f* [49]. In addition, one would expect higher contribution from the sample electrode interfacial polarization at low *f*, which is caused by blocking electrodes at the sample surfaces [50]. Drop in ε′ at high *f* is due to the -relaxation motion, which is active within this temperature range [7]. The -relaxation is ascribed to the long-range motions of chain segments within the crystalline–amorphous interphase [7,51]. In addition, some researchers link this relaxation to the micro-Brownian motions of chain segments within the amorphous phase [52].

PVDF/rGO and PVDF/TNL samples have lower ε′ values when compared to the unfilled PVDF. This trend is perhaps due to the agglomeration of the relatively more conductive fillers (rGO and TNL) which was confirmed by the TEM in Figure 5. Agglomeration of the nanoparticles would cause trapping of charges at their interfaces due to the Maxwell–Wagner–Sillars (MWS) interfacial polarization effect [53], whereas the hybrid PVDF/rGO-TNL exhibits higher ε′ due to the good dispersion of rGO and TNL within the PVDF matrix. Well dispersed material reduces the traps of charges within interfaces and form a good conductive composite. The hybrid PVDF/rGO-TNL composite has the highest polarizable components, which means higher ability to store electrical charges and could be useful as a supercapacitor material [54,55]. For example, the dielectric constant of the hybrid composite at 200 Hz is 3.6 times higher than that of the unfilled PVDF.

**Figure 15:**
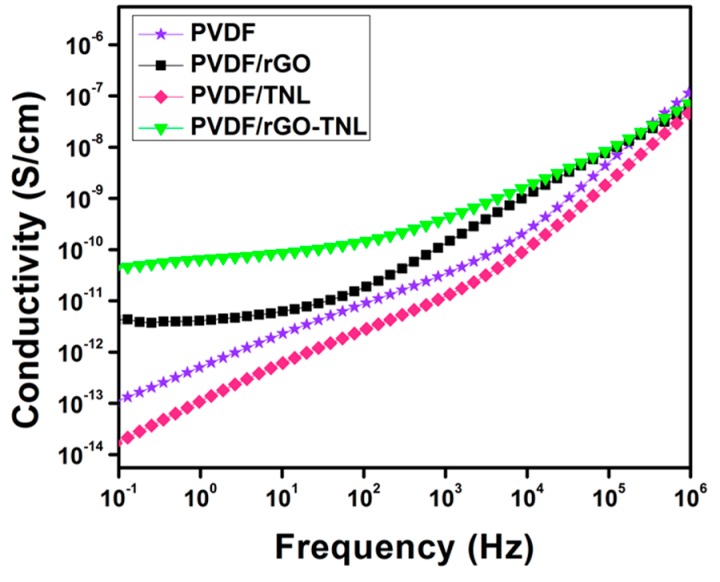
Conductivity vs. *f* at 20 °C for the PVDF composite samples with various nanofillers.

An indicator of the potential of these nanocomposites as good candidates for energy storage systems can be observed in Figure 14a,b, which illustrates the high ε′ relative to ε″ values for all samples, as indicated by the BDS spectra at 20 °C. This indication is further supported by the samples conductivity vs. *f* behavior shown in Figure 15. Both PVDF/rGO-TNL and PVDF/rGO composites have higher conductivity comparing to other samples as indicated by the vertical upshifts and presence of plateau regions at low *f*. The maximum electrical energy (*U*_max_) that can be stored in a linear dielectric material is given by Equation (5).

(5) $$U_{\max}=\;\varepsilon \prime {E_{b}}^{2}/2$$

where *E_b_* is the dielectric breakdown strength (DBS). For efficient electric energy storage, high DBS and ε′ are required [54].

### 3.6. Pressure Sensing

**Figure 16:**
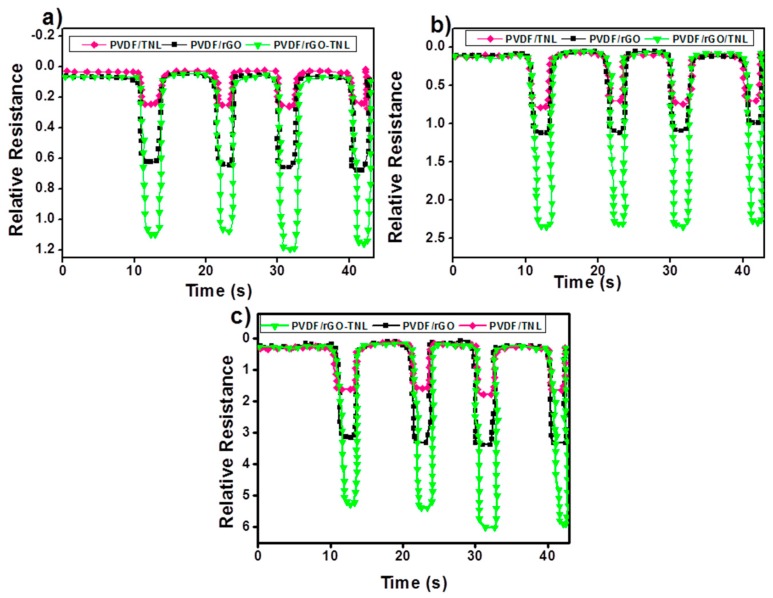
Sensing responses of PVDF/rGO, PVDF/TNL and PVDF/rGO-TNL composites to: (**a**) 5 kPa; (**b**) 10.7 kPa; and (**c**) 17.6 kPa.

The relative resistance variation of flexible PVDF/rGO-TNL, PVDF/TNL, and PVDF/rGO composites, for the first four cycles, is illustrated in Figure 16. PVDF/rGO-TNL has the highest relative resistance compared to other composites for all the three applied pressures. For each composite, upon pressure, the relative resistance decreased immediately. Tunneling is the common mechanism by which the electric current move between the electrodes in pressure sensing [15]. The compression of the sample (reduction in thickness) reduces the tunneling barriers and a tunneling path can be formed which leads to reduction in the resistivity of the composite. TNL effect is lower than rGO in improving the tunneling (for example the relative resistance reduced by 334.7% and 70.27% respectively at a pressure of 5 kPa). The agglomeration of nanoparticles in the matrix (while using one type of additive) does not allow formation of conducting paths. When pressure is applied to the sample, the agglomerated additives follow the movement of the polymer chains and cause forming conducting networks of the sample. The largest improvement in the hybrid composite can be attributed to the less agglomeration in the matrix and better dispersion. The sensitivity of hybrid composite increased by 333.46% at 5 kPa, 200.7% at 10.7 kPa and 246.7% 17.6 kPa compared to the PVDF/TNL.

**Figure 17:**
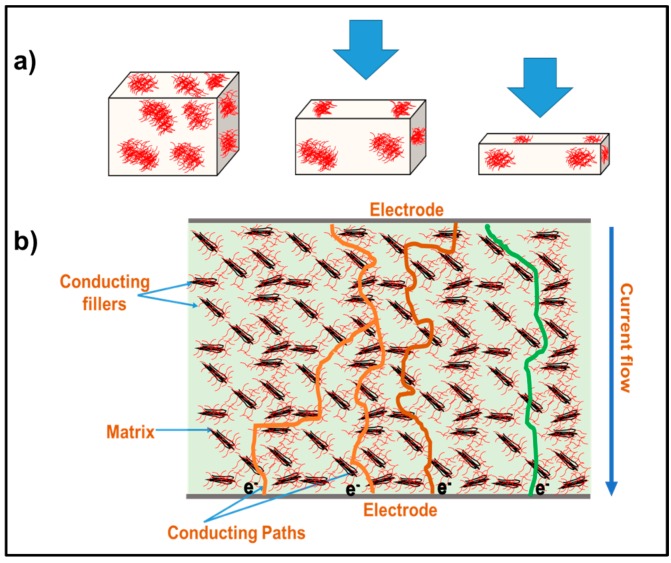
Schematic of: (**a**) behavior of TNL agglomerates under applied compression force, additives exhibit higher inter-distance after compression; and (**b**) conducting network formation between TNL, rGO and TNL-rGO particles.

The morphology of the additives plays a major role in improving the tunneling effect. The influence of pressure on the conducting networks present within the composite is schematically represented by Figure 17a. With the application of pressure, the well distributed nanolayers come closer and thereby decreasing the resistance of the sample. These conducting networks can be formed among the different conducting particles like rGO and TNL within the medium particularly in the case of the well dispersed hybrid composite, PVDF/rGO-TNL. This aspect is schematically demonstrated in Figure 17b. The presence of sharp and nanostructured tips can also improve the tunneling [56] as shown in the same figure. Thus, the interaction between the TNL and rGO with “sharper” morphology can increase the tunneling effect, thereby enhancing the magnitude of relative resistance change offers better pressure detection. Tuning the surface morphology of the nanofillers and enhancing the filler-polymer compatibility by regulating the preparation methods for enabling low-pressure detection will be the subject of our next communication.

## 4. Conclusions

A flexible PVDF/rGO-TNL pressure sensor was prepared by solution casting method. The flexible material showed good sensing properties with high response in short time. This performance was attributed to the interaction between TNL and rGO in the matrix and the well dispersed hybrid additive. Conducting networks were initiated during applying pressure, and improved by the sharp edges of the additives. The aggregation decrease in the hybrid composite of PVDF/rGO-TNL compared to PVDF/rGO and PVDF/TNL was confirmed by SEM, TEM and AFM studies. The good mechanical strength for PVDF/rGO-TNL was observed as a higher storage modulus was indicated for a novel hybrid composite, which is linked to the presence of β crystalline phase and reduction in α phase. The good dispersion improved the dielectric properties and increased the smoothness and wettability, which can reduce the bio-fouling which is highly needed in many sensing applications.

## Figures and Tables

**Table 1:** Composition of the composite samples.

Samples	Concentration of rGO (wt %)	Concentration of TNL (wt %)	Water Contact Angles (°)
PVDF	-	-	99.7 ± 4.2
PVDF/rGO	5	-	95.5 ± 3.2
PVDF/TNL	-	5	98.1 ± 3.1
PVDF/rGO-TNL	2.5	2.5	114.5 ± 3.5

**Table 2:** Tensile test results of PVDF/rGO-TNL, PVDF-TNL, PVD-rGO and PVDF.

Samples	Tensile Strength (MPa)	Young’s Modulus (MPa)	Elongation at Break (%)
PVDF	21.825 ± 1.93	1365.50 ± 101.23	16.22 ± 1.45
PVDF/rGO	22.927 ± 1.197	2969 ± 380.6	7.352 ± 0.66
PVDF/TNL	41.53 ± 1.58	3112.7 ± 173.60	5.40 ± 0.56
PVDF/rGO-TNL	46.91 ± 0.99	5010.65 ± 243.35	4.01 ± 0.49

**Table 3:** DSC data of PVDF/rGO-TNL, PVDF/rGO, PVDF/TNL, and PVDF.

Sample	Crystallization Temperature (°C)	Melting Temperature (°C)	∆*H*_m_ (J/g)	Degree of Crystallinity (*X*_c_, %)
PVDF	137.16	167.33	48.5392	46.227
PVDF/rGO	140.91	169.19	47.4923	45.23
PVDF/TNL	143.49	170.27	43.0843	41.03
PVDF/rGO-TNL	142.1	170.63	40.2879	38.36
